# Design and application of a novel two-amplicon approach for defining eukaryotic microbiota

**DOI:** 10.1186/s40168-018-0612-3

**Published:** 2018-12-20

**Authors:** Ana Popovic, Celine Bourdon, Pauline W. Wang, David S. Guttman, Wieger Voskuijl, Michael E. Grigg, Robert H. J. Bandsma, John Parkinson

**Affiliations:** 1 Program in Molecular Medicine, The Hospital for Sick Children, Peter Gilgan Centre for Research and Learning, 686 Bay Street, Toronto, ON M5G 0A4 Canada; 2 Department of Biochemistry, University of Toronto, Medical Sciences Building, 1 King’s College Circle Suite 5207, Toronto, ON M5S 1A8 Canada; 3 Program in Translational Medicine, The Hospital for Sick Children, Peter Gilgan Center for Research and Learning, 686 Bay Street, Toronto, ON M5G 0A4 Canada; 4 The Childhood Acute Illness & Nutrition Network (CHAIN), Nairobi Coordination Centre, P.O Box 43640-00100, 197 Lenana Place 2nd Floor, Nairobi, Kenya; 5 Department of Cell and Systems Biology, University of Toronto, 25 Harbord St, Toronto, ON M5S 3G5 Canada; 6 Centre for the Analysis of Genome Evolution and Function, University of Toronto, 25 Willcocks St Suite 4038, Toronto, ON M5S 3B2 Canada; 7 Department of Pediatrics and Child Health, the College of Medicine, University of Malawi, Mahatma Gandhi, Private Bag 360, Chichiri, Blantyre, Malawi; 8 Global Child Health Group, Emma Children’s Hospital, Academic Medical Center, Meibergdreef 9, 1105 AZ Amsterdam, The Netherlands; 9 Molecular Parasitology Section, Laboratory of Parasitic Diseases, NIAID, National Institutes of Health, 5601 Fishers Lane, MSC 9806, Bethesda, MD 20892-9806 USA; 10 Division of Gastroenterology, Hepatology and Nutrition, Hospital for Sick Children, 525 University Avenue, Toronto, ON M5G 2L3 Canada; 11 Centre for Global Child Health, Hospital for Sick Children, 525 University Avenue Suite 702, Toronto, ON M5G 2L3 Canada; 12 Department of Nutritional Sciences, University of Toronto, Medical Sciences Building, 1 King’s College Circle Suite 5253A, Toronto, ON M5S 1A8 Canada; 13 Department of Biomedical Sciences, College of Medicine, University of Malawi, Mahatma Gandhi, Private Bag 360 Chichiri, Blantyre, Malawi; 14 Department of Molecular Genetics, University of Toronto, Medical Sciences Building, 1 King’s College Circle Suite 4386, Toronto, ON M5S 1A8 Canada

**Keywords:** Microbiome, Eukaryotes, Parasites, Next-generation sequencing, Amplicon sequencing, Malnutrition

## Abstract

**Background:**

Due to a lack of systematic diagnostics, our understanding of the diversity and role of eukaryotic microbiota in human health is limited. While studies have shown fungal communities to be significant modulators of human health, information on the prevalence of taxa such as protozoa and helminths has been limited to a small number of species for which targeted molecular diagnostics are available. To probe the diversity of eukaryotic microbes and their relationships with other members of the microbiota, we applied in silico and experimental approaches to design a novel two-amplicon surveillance tool, based on sequencing regions of ribosomal RNA genes and their internal transcribed spacers. We subsequently demonstrated the utility of our approach by characterizing the eukaryotic microbiota of 46 hospitalized Malawian children suffering from Severe Acute Malnutrition (SAM).

**Results:**

Through in silico analysis and validation on a diverse panel of eukaryotes, we identified 18S rRNA variable genetic regions 4 and 5 (*18S V4 V5*), together with a region encoding 28S rRNA variable genetic region 2 and the internal transcribed spacers (*transITS*), as optimal for the systematic classification of eukaryotes. Sequencing of these regions revealed protozoa in all stool samples from children with SAM and helminths in most, including several eukaryotes previously implicated in malnutrition and diarrheal disease. Clinical comparisons revealed no association between protozoan parasites and diarrhea or HIV reactivity. However, the presence of *Blastocystis* correlated with bacterial alpha diversity and increased abundance of specific taxa, including *Sporobacter*, *Cellulosibacter*, *Oscillibacter*, and *Roseburia*.

**Conclusion:**

We suggest this novel two-amplicon based strategy will prove an effective tool to deliver new insights into the role of eukaryotic microbiota in health and disease.

**Electronic supplementary material:**

The online version of this article (10.1186/s40168-018-0612-3) contains supplementary material, which is available to authorized users.

## Background

Microbial communities in the human body are significant modulators of health and disease. The contributions of gut bacteria have been well documented; however, excluding fungi [1], little is known concerning the diversity and role of eukaryotic microbiota. Studies relying on targeted molecular diagnostics, such as PCR and ELISA or microscopy, have revealed the presence of several protozoa, including both commensal and parasitic species, in the gut of healthy individuals. For example, *Blastocystis* was present in stool from 56% of 105 Danish adults and all of the 93 samples obtained from Senegalese children [2, 3]. Furthermore, *Dientamoeba fragilis* has been found in 68% of children in a Danish daycare, while *Giardia* was identified in 8% of children attending Toronto daycares and 32% of children sampled in rural Ecuador [4–6]. Although typically considered asymptomatic, *Blastocystis* and *Dientamoeba* have been linked with inflammatory bowel disease and irritable bowel syndrome [7–10], conditions that promote gut bacterial dysbiosis [11, 12]. Indeed, it is becoming apparent that bacteria and eukaryotic microbiota feature complex relationships both with each other and the host immune system; specific gut bacteria have been shown to confer protective immunity to the host against transmission of *Plasmodium* and *Toxoplasma gondii*, the etiological agents of malaria and toxoplasmosis respectively [13–16]. Conversely, induction of the host inflammasome by the protozoan *Tritrichomonas musculis* reduced *Salmonella* infection in mice, but exacerbated T cell-driven colitis [17].

Though more sensitive than microscopy or culture-based diagnosis [6, 18–20], molecular diagnostics are only available for a limited number of pathogens and provide no information on the diversity of eukaryotes in a defined environment. In attempts to better characterize the contribution of microbial eukaryotes to health, marker gene studies, analogous to bacterial 16S rRNA gene surveys, have been proposed. In these approaches, variable genetic regions are targeted for PCR amplification using universal DNA primers which bind to all organisms; sequencing of the resultant amplicons subsequently yields a readout of taxa present [21–24]. In one such study, amplicons generated from the 18S rRNA gene revealed protozoa in stool from all 23 Malawian individuals sampled [25]. However, such PCR-based approaches are not without challenges. The enormous sequence diversity exhibited by eukaryotes can result in established primers failing to amplify regions from entire phyla [22, 24]. Conversely, high sequence conservation in the region being targeted can limit resolution at the level of species or genus.

To address these limitations, we present a two-amplicon approach to generate a robust profile of eukaryotic microbiota. We perform a systematic in silico analysis to select two candidate amplicons suitable for classification of eukaryotic microbes: (1) the 18S rRNA variable genetic regions 4 and 5 (V4 V5) and (2) a region encompassing the internal transcribed spacers (ITS), together with the 18S rRNA gene V9 and the 28S rRNA gene V1 V2, collectively referred to as *transITS*. We design and validate universal DNA amplicon primers for these two regions using a diverse panel of eukaryotes. We subsequently demonstrate the effectiveness of our approach to survey eukaryotic microbiota in a study of stool samples from 46 Malawian children hospitalized for Severe Acute Malnutrition (SAM) [26, 27], a cohort with a high expected prevalence of intestinal parasites. We show that in addition to fungi, protozoa and helminths are prevalent in this cohort. We identify associations between protozoan *Blastocystis* and bacterial taxa, but find no associations between gut parasites and clinical features, including HIV reactivity and diarrhea.

## Results

### Amplicon identification and primer design

**Fig. 1:**
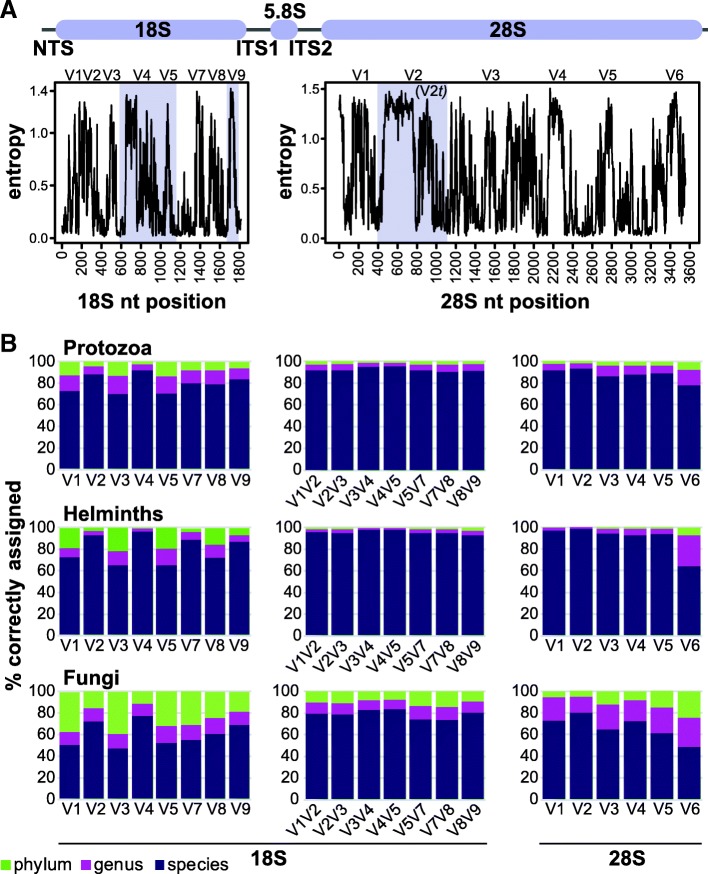
Identification and evaluation of biomarker regions in 18S and 28S rRNA genes. **a** Sequence variability of protozoan 18S and 28S rRNA genes. Variability was calculated as the Shannon entropy at each position along aligned protozoan sequences. Identified variable regions are numbered and those selected for further analysis are highlighted in purple. The graphic at the top depicts the canonical arrangement of eukaryotic rRNA genes. **b** Accuracy of taxonomic classification of variable regions to the phylum, genus, and species levels. Taxonomies of 100 copies of variable regions in each gene, randomly mutated with 1% frequency, were compared to the best match identified by BWA-MEM in the SILVA v128 database. Results are shown for protozoan (19,427 18S; 1061 28S), helminth (3882 18S; 1237 28S), and fungal (14,657 18S; 3625 28S) rRNA genes

We explored the sequence variability in eukaryotic rRNA genes in the SILVA v128 database to identify taxonomically informative genetic regions. Shannon entropy calculations of aligned genes revealed eight highly variable genetic stretches in the 18S rRNA gene and six in the 28S rRNA gene (Fig. 1a). 18S rRNA variable genetic regions (V) are named according to convention, and the number identified agrees with previously published data for eukaryotes [21, 22].

In silico analyses were performed to evaluate the ability of each region to correctly classify taxa. For each variable region, 100 copies of each representative sequence were generated with a 1% mutation rate introduced to simulate sequencing error. Based on taxonomies of top hits in a sequence comparison with genes from SILVA, 18S rRNA gene V4 and 28S rRNA gene V2 achieved the highest accuracies in identifying protozoan (91.7%, 92.9%) and helminth (96%, 98.5%) species (Fig. 1b; Additional file 1: Table S1). Extending 18S rRNA gene amplicons to include two variable regions increased accuracy to 94.9% and 97.5% respectively, in the V4 V5 region. Identification of fungal species, by comparison, achieved a maximum accuracy of 83%, highlighting the reliance on ITS1 and ITS2 sequences for fungal studies. Based on these results, we selected 18S rRNA gene V4 V5 and 28S rRNA gene V2 for DNA primer design, as well as the Earth Microbiome Project (EMP) recommended 18S rRNA gene V9 [28].

**Fig. 2:**
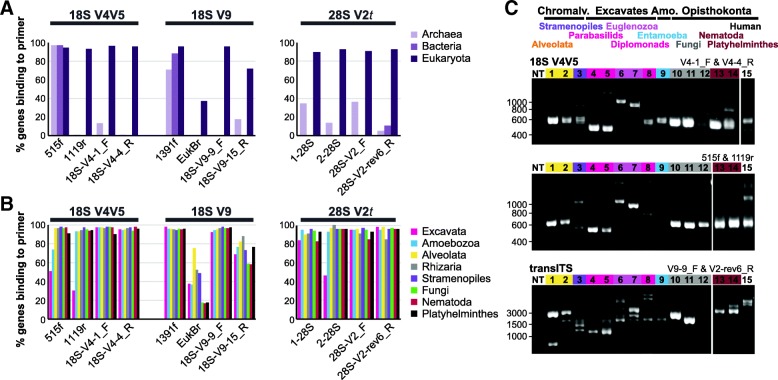
In silico and experimental evaluation of amplicon primers. Predicted DNA primer binding **a** to eukaryote, archaeal, and bacterial genes and **b** to protozoan and helminth kingdoms. Primers used in previous published 18S rRNA gene sequencing studies and the highest scoring primers designed for this study are shown for three potential amplicon regions. Tests were performed using SILVA TestProbe3.0 against the v128 Ref_Nr99 (18S) and Ref (28S) databases. **c** PCR amplification of the V4 V5 amplicon from the 18S rRNA gene using V4-1_F and V4-4_R, or 515f and 1119r primers (top), and the transITS amplicon using V9-9_F and V2-rev6_R primers (bottom). Reactions include a no template control, and template genomes from (1–15) *T. gondii* RH, *N. caninum* [NcBahia], *B. hominis*, *T. vaginalis* SD7, *T. muris* G1, *T. cruzi* [DA], *L. panamensis* [WR470], *G. intestinalis* [G3M], *E. dispar*, *S. cerevisiae*, *C. albicans*, *A. fumigatus*, *C. elegans*, *S. mediterranea*, and a human foreskin fibroblast cell line (ATCC SCRC-1041)

Based on comparisons of sequence conservation profiles across the following clades: Stramenopiles, Alveolata, Rhizaria, Excavata, Amoebozoa, Haptophyta, Cryptophyta, Platyhelminthes, and Nematoda, we designed 12, 19, and 16 candidate primers targeting 18S rRNA gene V4 V5 and V9, and the 28S rRNA gene V2, respectively (Additional file 1: Table S2). Each primer, together with 43, 21, and 3 previously published primers targeting the same regions [21–24], were evaluated in silico using the SILVA TestProbe 3.0 tool [29]. In terms of taxonomic coverage, 18S rRNA gene V4 V5 primers V4-1_F and V4-4_R generally produced the best results, (Fig. 2a, b; Additional file 1: Table S2). V4-1_F and published primer 574′f achieved coverage within 1% for all kingdoms, with V4-1_F performing better for Amoebozoa, Alveolata, and Nematoda, and 574′f for Excavata, Rhizaria, and Platyhelminthes. We predict that the absence of degenerate nucleotide positions in V4-1_F compared to three in 574′f will reduce spurious PCR products. By contrast, 515f and 1119r, previously used in an 18S survey of eukaryotes [25], are predicted to bind 65% fewer Excavata and 24% fewer Amoebozoa sequences (Fig. 2b).

Focusing on the 18S rRNA gene V9, no single primer outperformed others across all clades. While 1391f is predicted to bind more Excavata, Amoebozoa, and Alveolata genes, V9-9_F performed better for Stramenopiles, Rhizaria, Nematoda, and Platyhelminthes, with an additional advantage of low predicted binding to bacterial rRNA genes, reducing contamination. The accuracy of predicted reverse primer binding was likely compromised by incomplete reference genes. For the 28S rRNA gene V2 region, we found that lack of conservation at the 5′ end of this region resulted in the loss of 80% of genes from Excavata. Instead, we designed a primer to bind *within* V2 (28S V2_F). Primers V2_F and V2-rev6_R are predicted to bind up to 50% more Excavata and 6% more Alveolata sequences, at a 15% reduction in Rhizaria, compared to published primers (Fig. 2b). The resulting truncated amplicon (V2 *t*) is ~ 50% shorter (Fig. 1a), however, limiting taxonomic assignment.

Given the limitations of the reverse 18S rRNA gene V9 and forward 28S rRNA gene V2 primers, we explored the possibility of generating a larger amplicon (“*transITS*”) using the 18S V9-9_F and 28S V2-rev6_R primers. The transITS benefits from sequence variability of the entire 28S rRNA gene V2 and ITS previously used for strain typing. Amplification of ITS regions alone is not feasible for protozoa due to high variability in 5.8S and 5′ of 28S rRNA genes. Given ITS hypervariability and paucity of protozoan reference sequences, we reasoned that 18S rRNA gene V9 and 28S rRNA gene V2 could provide high-level taxonomic information, and where available, ITS regions could resolve species or strains.

### Primer validation

Based on our in silico analyses, we validated the following primer pairs on a diverse panel of eukaryotes that include nine protozoa, three fungi, two helminths, and cultured human cells: 515f and 1119r, V4-1_F and V4-4_R, and 18S V9-9_F and 28S V2-rev6_R. For the 18S rRNA gene V4 V5 region, our V4-1_ F and V4-4_R primers were successful across the entire panel with the exception of the fungus *A. fumigatus*, which produced only a weak PCR product. On the other hand, the 515f and 1119r primer pair showed poor amplification of *Blastocystis*, *Entamoeba*, and *Giardia* (Fig. 2c). As for our 18S V4 V5 primer set, transITS amplicons were generated across the panel with the exception of *A. fumigatus* (Fig. 2c). As this amplicon can reach lengths of several thousand bases in select taxa such as *Sarcocystis*, we employed a processive high fidelity polymerase and 3-min extension times to maximize amplification (e.g., as shown with the successful recovery of the ~ 4000 bp human transITS region).

### 18S rRNA gene V4 V5 amplicon surveys reveal eukaryotic microbiota in children with SAM

We applied a two-amplicon approach to investigate the eukaryotic microbiota in stool from Malawian children hospitalized with complicated SAM [26, 27]. Due to limited DNA, we amplified sufficient 18S rRNA gene V4 V5 and transITS amplicons from 44 and 46 samples, respectively. Clinical features of the children are detailed in Table 1.

**Table 1:** Characteristics of patients hospitalized for severe acute malnutrition

	All		Edematous SAM		Severe wasting		*p*
	*n* = 44		*n* = 27		*n* = 17		
Female, *n* (%)^1^	25*	(57%)	15	(55%)	10	(59%)	1
Age, months	23	± 11.8	27.1	± 11.7	17.9	± 9.8	.02
Length, cm	75.9	± 9.0	79.8	± 7.4	69.8	± 8.0	.002
Height-for-age, *Z*-score	− 3.0	± 2.0	− 2.6	± 1.9	− 3.7	± 1.9	.08
Weight-for-age, *Z*-score	− 3.7	± 1.7	− 2.9	± 1.3	− 5.1	± 1.1	< .001
Weight-for-height, *Z*-score	− 3.0	± 1.8	− 2	± 1.4	− 4.6	± 1.0	< .001
Diarrhea, *n* (%)^2^	15	(36%)	11	(40%)	4	(27%)	.5
HIV reactive, *n* (%)^3^	18*	(45%)	7	(29%)	11	(69%)	.02
Death^4^	7	(16%)	2	(7%)	5	(29%)	.09

^1^Two children are of unknown sex

^2^Four have unknown diarrhea

^3^Six have unknown HIV reactivity (edematous SAM, *n* = 3; severe wasting, *n* = 3)

^4^four have unknown death status

**Fig. 3:**
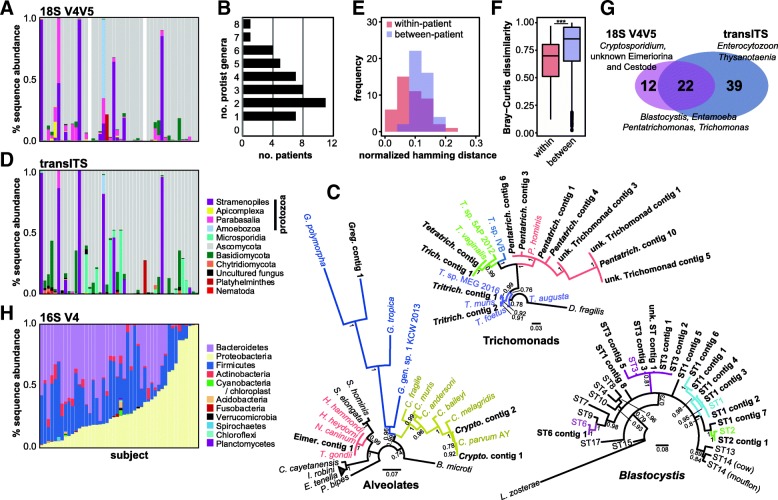
Eukaryotic and bacterial microbiota of Malawian cohort suffering from SAM. **a** Relative sequence abundance of microbial eukaryotic phyla, identified by the 18S rRNA gene V4 V5 amplicon (*n* = 44). Columns represent individual patients. **b** Numbers of protozoan genera identified in patients from 18S rRNA gene V4 V5 data. **c** Bayesian phylogenetic trees showing relationships between sequence representatives from clinical samples and reference species. Posterior probability values indicate branch support. **d** Relative sequence abundance of microbial eukaryotic phyla, identified by transITS (*n* = 46) amplicons. **e** Similarity of taxonomic profiles generated by the two amplicon regions, quantified by co-occurrence of eukaryotic genera *within* patients compared to *between* patients. Shown are distributions of within-patient and between-patient normalized Hamming distances, averaged over a range of presence/absence thresholds (2–100 reads). **f** Bray-Curtis compositional dissimilarity of eukaryotic genera identified by the two amplicon regions *within* and *between* patients. Significance was tested using the Wilcoxon rank sum test. **g** Venn diagram showing the overlap of eukaryotic genera identified using the two amplicon regions. A subset of organisms of interest is indicated. **h** Relative sequence abundance of bacterial phyla identified in 16S rRNA gene amplicon data

Sequencing of the 18S rRNA gene V4 V5 amplicon generated from 22,091 to 193,949 quality-filtered sequences per sample (see Additional file 1: Table S3). Taxonomic classification revealed varying proportions of vertebrate (0.03–95%), plant (0–90%), fungal (0.5–100%), protozoan (0.01–99%), and helminth (0–20%) sequences (Fig. 3a; Additional file 2: Figure S1). Protozoan sequences were identified in all children, with 37 (84%) carrying at least two genera (Fig. 3b). Twelve genera from five phyla were found in total. Protozoa detected with the highest relative sequence abundance include *Blastocystis spp.* (98%), *Entamoeba coli* (64%), and *Pentatrichomonas hominis* (54%). The most prevalent, found in > 80% of children, were *Trichomonas* and *Tritrichomonas* (Additional file 1: Table S4), while amplicon sequences classified to the suborder Eimeriorina were detected in 24 samples at ≤ 2% abundance. A previously undescribed trichomonad organism was also detected in two samples, at 6% and 15% relative abundance. *Blastocystis* amplicons represent multiple subtypes which are known to differ in prevalence and exhibit substantial genetic diversity [30]. Reflecting previous studies [25, 31, 32], we found mixed *Blastocystis* colonization. The most prevalent subtypes, defined by > 98% sequence identity, were ST3, followed by ST1, ST2, and ST6 (Additional file 1: Table S4). Interestingly, *Cryptosporidium* was identified in only two samples using the commercial Luminex Gastrointestinal Pathogen Panel [26], while we detected it in an additional 10 samples using 18S rRNA gene V4 V5 amplicon sequencing. This may suggest species variants not captured by the Luminex diagnostic. In addition to protozoa, we detected three helminths, including a Cestode (tapeworm) at > 20% relative abundance in one sample. Most identified fungi were Ascomycota, ranging from 0.5 to 100% relative abundance, dominated by *Candida* and other *Saccharomycetales*. Through targeted PCR, we verified the presence of *Pentatrichomonas* and *Entamoeba coli*, as well as *Cryptosporidium* in at least four samples, using published primers and one primer designed for this study (Additional file 2: Figure S1).

In an attempt to verify taxonomic assignments based on 200 nt of the high-quality amplicon forward reads, we incorporated > 100 nt of reverse read information and used a binning and assembly approach to undertake more robust phylogenetic analyses for three major protozoan clades (Alveolates, Trichomonads, and *Blastocystis*; Fig. 3c). In brief, read pairs assigned to each clade were binned into discrete genera. Each genus bin was then assembled resulting in a total of 336 contigs. After merging contigs sharing ≥ 99% sequence identity, we recovered 27 contigs generated from at least 100 read pairs (3, 11, and 13 for Alveolates, Trichomonads, and *Blastocystis* respectively). To expand representatives in each clade, we additionally included four contigs associated with *Gregarina*, *Tetratrichomonas*, and *Blastocystis* sp. ST6 and unknown ST (generated from 32, 26, 56, and 23 read pairs respectively). Together with sequences from representative organisms, these contigs were used to construct three clade-specific phylogenies. Both Trichomonad and Apicomplexan phylogenies were consistent with amplicon assignments. The position of Eimeriorina contig 1 suggests these amplicons derive from a member of the *Toxoplasmatinae*, members of which are challenging to resolve at this locus. Contigs assembled from amplicons previously classified as “unknown Trichomonad” appear most closely related to *Pentatrichomonas*, while amplicon sequences from the *Tetratrichomonas* contig appear to have been misassigned due to close sequence similarity with *Trichomonas* in the 200 nt classified region. The majority of *Blastocystis* amplicon sequences were classified to the correct subtype, with the exception of ST1 contigs 5, 7, and 8, collectively representing 762 amplicon sequences. With additional sequence information, we identified amplicon sequences classified as “unknown subtype” as ST3.

We note that while the Luminex diagnostic identified *Giardia* and *Entamoeba histolytica* in 13 and 1 samples, respectively, both taxa were missing from our amplicon analyses. We did detect *Giardia* and a close relative, *Entamoeba dispar*, during primer validation tests (Fig. 2c). It may be that the abundance of *E. histolytica* is below the sensitivity of our system. Primer mismatches to *Giardia* rRNA genes may limit amplification in the presence of competing sequences, a limitation also noted for 515f and 1119r primers [25].

### TransITS amplicon surveys complement findings from the 18S rRNA gene V4 V5 amplicon

TransITS amplicons have lengths up to several thousand bases, and include segments of the 18S and 28S rRNA genes, the 5.8S rRNA gene, and ITS regions. For Illumina sequencing, these amplicons were randomly fragmented to median 300 bp using the Nextera DNA library kit. Sequencing generated 44,750 to 192,224 quality-filtered reads per sample (see Additional file 1: Table S3). Reads (≥ 100 nt) were classified by sequence similarity to a custom reference database using the BWA-MEM aligner [33], followed by a BLAST search of unclassified sequences against the NCBI nucleotide database, using ≤ 3% nucleotide mismatch and ≥ 70% sequence coverage. We identified eukaryotes, bacteria, a small number of archaeal sequences, and over 1.1 million unclassified sequences out of 5.3 million (0.1 to 54% per sample). Among eukaryotes, we found eight protozoan genera, a microsporidian, six helminths, and six phyla of fungi (Fig. 3d; Additional file 1: Table S4). *Pentatrichomonas hominis* and *Entamoeba coli* were detected in the same patients as found by 18S rRNA gene V4 V5 data, and *Blastocystis* was found in 12 patients, with similar patterns of abundance. *Trichomonas* and *Tritrichomonas* were detected at low abundance in three and two samples, respectively. While we did not detect *Cryptosporidium* using this amplicon, interestingly, we identified microsporidian parasite *Enterocytozoon bieneusi* in 12 samples. Helminth sequences were classified primarily as *Thysanotaenia congolensis* isolate CY7 in one sample, for which no 18S rRNA gene V4 V5 data was generated. The most abundant fungal genera were *Candida*, *Nakaseomyces*, *Trichosporon*, *Kazachstania*, *Pichia*, *Clavispora*, and *Saccharomyces*.

We evaluated the similarity between the profiles of eukaryotic microbiota generated by the 18S rRNA gene V4 V5 and transITS amplicons through taxon co-occurrence using Hamming distance comparisons and Bray-Curtis dissimilarity. We find a higher co-occurrence of genera and phyla in pairs of amplicon datasets from the same patient compared to between patients (*p* values 8E−6 and 0.006) (Fig. 3e). Bray-Curtis analysis including taxa detected by both amplicon approaches found significantly higher compositional similarity between 18S rRNA gene V4 V5 and transITS profiles from the same patients (*p* value 1.57E−05) (Fig. 3f). Indeed, the two amplicons share six of the top 10 abundant genera. However, no significant associations were found when all taxa were included, consistent with previous studies that compared multiple amplicons [34, 35]. Using our two-amplicon approach, we were able to detect an additional 39 genera to those identified by 18S rRNA gene sequencing alone, while the 22 genera captured by both amplicons represent a high-confidence set of protozoa, helminths, and fungi identified in Malawian SAM patients (Fig. 3g). This demonstrates the potential to sample a broader range of taxa, circumventing amplicon-specific biases, and to cross-validate organisms identified by two sequencing experiments.

**Fig. 4:**
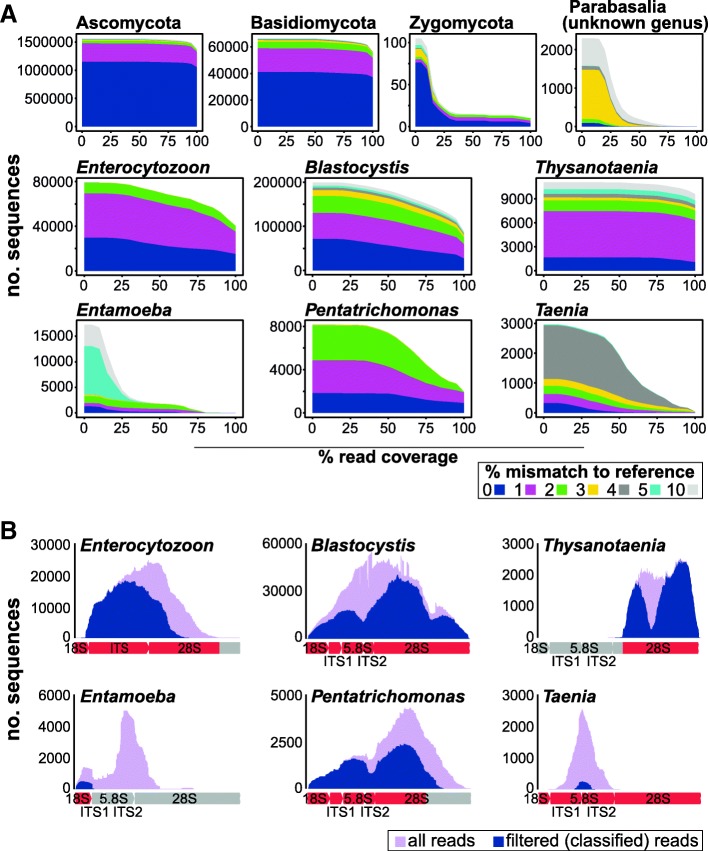
Classification profiles of the transITS amplicon. **a** Graphs show numbers of sequences (*y*-axis) classified to indicated eukaryotic taxa, at various minimum sequence coverage thresholds (*x*-axis). Colors indicate the maximum percent mismatch between amplicon and reference sequences. **b** Read coverage depicted across positions of the amplicon. Shown are total numbers of reads (violet) and reads with ≥ 97% sequence identity across ≥ 70% of the read length (blue) classified to a genus. Graphics below plots indicate available (red) or missing (gray) reference sequences for the genus

Relative to the 18S rRNA gene locus, the transITS region has more limited taxonomic representation in sequence databases. We were therefore interested in examining the ability of reference sequences to capture sequence diversity encountered in our samples. We first considered how many amplicon reads shared sequence similarity with reference sequences at various thresholds of sequence identity and read coverage (Fig. 4a). The majority of fungal reads were ≥ 99% identical to Ascomycota and Basidiomycota reference sequences suggesting good taxonomic representation in the reference database. On the other hand, reads classified to taxa such as *Enterocytozoon*, *Blastocystis*, *Pentatrichomonas*, *Thysanotaenia*, and *Taenia* reveal significant mismatches to reference sequences, suggesting they derive from species or strains not captured by the reference dataset. Low read coverage exhibited by taxa such as *Entamoeba* or Zygomycota also suggests incomplete or missing reference sequences.

As the transITS amplicons are fragmented for sequencing, we might expect a uniform sampling of reads from across the entire locus. To explore this, amplicon sequences were mapped to a representative reference sequence from the assigned species. Where no reference sequence was available, amplicon sequences were binned on the basis of species assignment and each bin assembled to form contigs. Contig consensus sequences were subsequently used as a reference. Interestingly, rather than displaying uniform coverage across the entire length of the amplicon, we find that amplicon sequences assigned to *Enterocytozoon*, *Blastocystis*, and *Pentatrichomonas* exhibit uneven coverage (Fig. 4b). For example, for *Blastocystis*, this is evident in the ITS2 region and 5′ of 28S rRNA gene. Other taxa, such as *Entamoeba* and *Taenia* exhibit limited representation across the locus. *Entamoeba* reads, for example, map only to the V9 region of the 18S rRNA gene, reflecting the low read coverage of *Entamoeba* sequences observed in Fig. 4a and lack of reference information for *Entamoeba coli* ITS and the 28S rRNA gene. Similarly, reference information is only available for the 28S rRNA gene for *Thysanotaenia*. Reads assigned with low scores to *Taenia* ITS and the 5.8S rRNA gene may belong to *Thysanotaenia* (Fig. 4b), as these were identified in the same sample. As genomes and sequences from clinical isolates are deposited into public databases, we predict that we will be able to classify more sequences with greater confidence.

Finally, we explored over 3000 unclassified reads with weak similarity to Parabasalids (Fig. 4a). Assembly of reads generated four contigs (> 10 reads), with the largest (995 nt long, containing 2103 reads) sharing 77% sequence identity over 62% of its length with the *Trichomonas vaginalis* 28S rRNA gene AF202181. Reads from this contig largely derive from two samples (1169 and 919 reads) in which our data from the 18S rRNA gene V4 V5 indicate the presence of an uncharacterized trichomonad. This likely suggests that the transITS reads derive from the same taxon; however, lack of DNA precludes further experimental validation.

### *Blastocystis* colonization is associated with changes in bacterial microbiota

To explore potential relationships between gut eukaryotes and bacteria, we characterized the bacterial communities through amplicon sequencing of the 16S rRNA gene. Sequencing generated 26,887 to 179,945 quality filtered reads per sample (Additional file 1: Table S3) and were dominated by Bacteroidetes, Firmicutes, and Proteobacteria (Fig. 3h). Proteobacteria represented > 20% of sequences in 26 out of 46 patients and > 80% in 6 patients. Of these, the most highly represented were *Escherichia-Shigella*, *Enterobacter*, *Pantoea*, and *Klebsiella*, genera known to include pathogenic members.

**Fig. 5:**
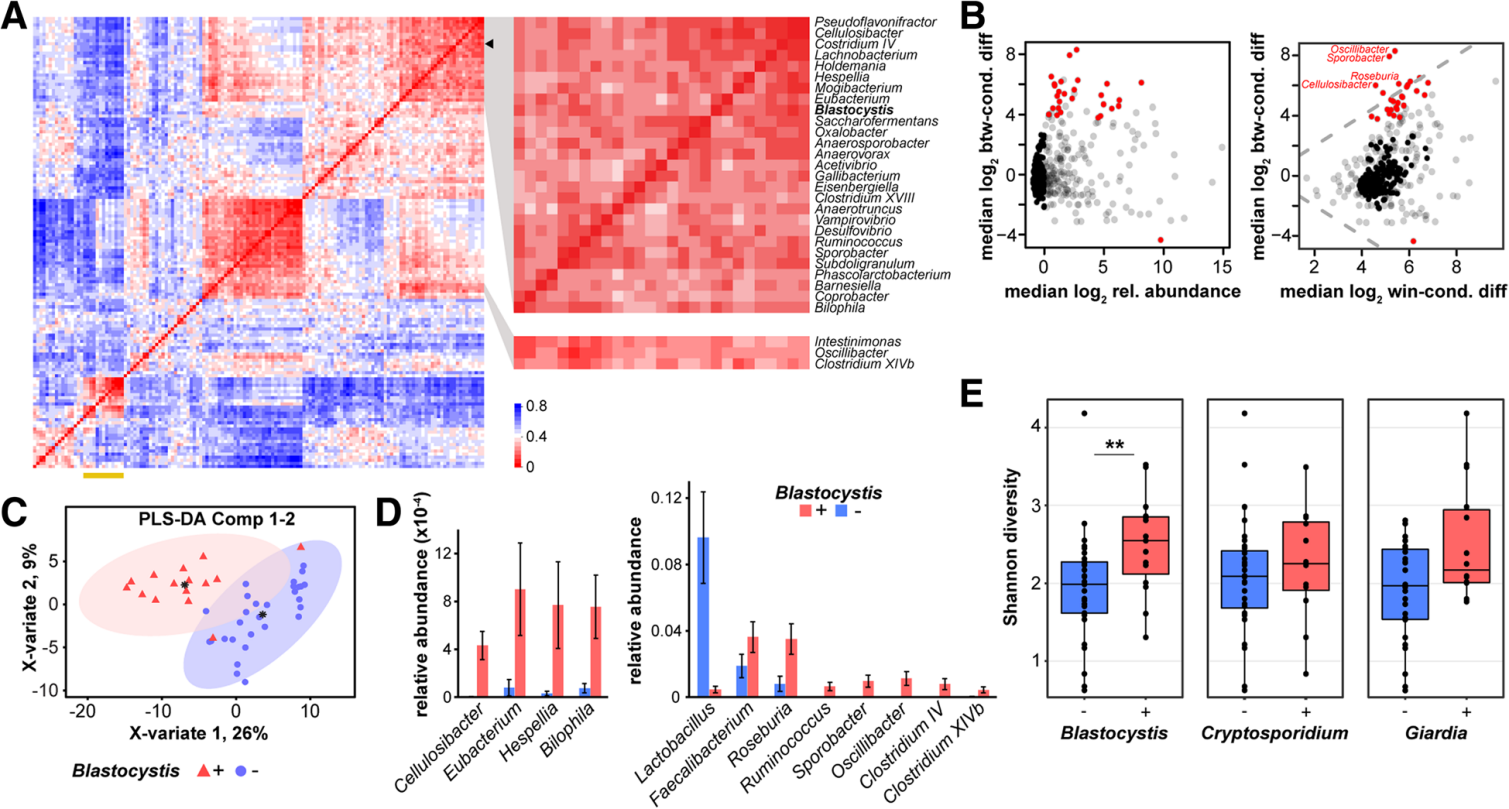
Associations between eukaryotic and bacterial microbiota. **a** Co-occurrence analysis between eukaryotic and bacterial microbiota. Shown is a heatmap of Hamming distances between genera represented by ≥ 5 reads in 20–80% of samples (*n* = 44). Red indicates a high co-occurrence score. The detail highlights bacterial genera clustering with *Blastocystis*, and the yellow line denotes a cluster of *Enterobacteriaceae*. **b** Association of bacterial composition with the presence of *Blastocystis*. Plots were generated with ALDEx2 and show OTU fold change versus median abundance (left) and OTU fold change between versus within conditions (right). Red points indicate OTUs with significant compositional changes (Wilcoxon rank test). **c** sPLS-DA analysis showing separation of patient samples based on the abundance of bacterial genera in *Blastocystis* positive and negative patients. **d** Mean relative sequence abundances of a subset of bacterial genera, in *Blastocystis* positive and negative patients. Error bars represent standard error. **e** Comparison of alpha diversity of samples positive and negative for *Blastocystis* (15; 29), *Cryptosporidium* (12; 32), and *Giardia* (14; 26), measured using the Shannon index. The presence of *Blastocystis* and *Cryptosporidium* were counted with a minimum of five reads. *p < 0.05, **p < 0.005

A co-occurrence analysis of bacterial and eukaryotic genera, represented as clustered Hamming distance scores, revealed blocks of associated taxa (Fig. 5a). We identified putative positive associations between *Blastocystis* and two Proteobacteria, *Bilophila*, and *Vampirovibrio*, together with a number of Clostridiales, notably *Cellulosibacter*, *Eubacterium*, *Sporobacter*, and *Hespellia*, as well as *Oscillibacter*, *Clostridium XIVb*, and *Intestinimonas*, present in a separate cluster. These findings are consistent with a previous study of a French cohort which reported similar associations with *Blastocystis* colonization [36]. Interestingly, the cluster containing *Blastocystis* appears to be negatively correlated with a group of *Enterobacteriaceae* (Fig. 5a).

Differential abundance analysis of bacterial OTUs using ALDEx2 revealed that only *Blastocystis* colonization was correlated with significant changes in 15 bacterial taxa confirming many co-occurrence associations, including the two Proteobacteria, *Bilophila*, and *Vampirovibrio* (Fig. 5b; Additional file 2: Figure S2; Additional file 1: Table S6). The most significant changes were associated with the Firmicutes *Oscillibacter*, *Sporobacter*, *Cellulosibacter*, and *Roseburia*. Notably, of the two butyrate producers *Faecalibacterium* and *Roseburia* previously correlated with *Blastocystis* [36], we were only able to confirm a significant association with *Roseburia*. sPLS-DA and relative abundance analyses of bacterial composition further corroborate our findings and show separation of patient samples based on *Blastocystis* colonization (Fig. 5 c, d). The first sPLS-DA component, driven by changes in *Sporobacter*, *Cellulosibacter*, *Oscillibacter*, *Eubacterium*, and *Lutispora* abundance, explained 20% of the variance between samples, with an estimated 9% classification error rate based on tenfold cross-validation. Bacterial richness and diversity analyses revealed that only *Blastocystis* colonization correlated with changes in bacterial population structure (*p* < 0.05 or *p* < 0.005; Fig. 5e; Additional file 1: Table S7). Whether *Blastocystis* modulates the growth and colonization of bacteria or is itself dependent on the presence of specific bacterial taxa requires further investigation.

### No evidence of association between parasite carriage, HIV reactivity, and diarrhea

Finally, we explored the relationships between clinical features of patients with SAM and the eukaryotic organisms *Blastocystis*, *Cryptosporidium*, *Giardia*, and *Enterocytozoon*. In our cohort, these eukaryotes showed no significant association with age, HIV reactivity, edema status, weight for height *Z*-scores (a measure of wasting), or with diarrhea (Additional file 1: Table S8). Similar results were obtained when including as appropriate age, sex, and HIV as cofounders in the models.

## Discussion

Despite the health and economic burden of a handful of intestinal protozoan and helminthic parasites worldwide, the diversity and role of these taxa in the microbiome is largely unexplored. Targeted surveys have revealed high carriage rates of a number of parasites in asymptomatic individuals, and studies are beginning to uncover key interactions between these organisms and gut bacteria [13, 37–42]. For example, treatment of mice with *Lactobacillus* and *Bifidobacterium* decrease *Plasmodium* burden [37], while gut colonization by *E. coli* 086:B7 and *E. coli* CFT073 protect against malaria transmission and *Cryptosporidium* infection, respectively [13, 41]. Helminth infections have been suggested to promote growth of protective Clostridiales, inhibiting colonization by an inflammatory Bacteroides species [42]. To better study these relationships, a broad surveillance diagnostic is needed. In an attempt to overcome limitations such as primer bias and poor taxonomic resolution associated with existing biomarkers in the 18S rRNA gene, we designed a two-amplicon approach, based on 18S and 28S rRNA genetic regions and internal transcribed spacers. By combining two regions, we report a high confidence set of taxa identified by two independent diagnostics and the potential to capture greater protozoan and helminth diversity in a pilot cohort of SAM patients, as well as uncover interactions with gut bacteria.

Our identification of amplicon V4 V5 in the 18S rRNA gene builds on CBOL Protist Working Group recommendations for the use of the 18S rRNA V4 genetic region for broad eukaryotic surveys [43] and previous ecological surveys [44–46]. Previous studies have shown that sequencing two amplicon regions in this gene provides complementary information on eukaryotic microbes [45, 47]. Here, we extend this approach by combining data from the 18S rRNA gene with that from the ITS regions and the 28S rRNA gene in our transITS amplicon. The ITS region is currently used for strain typing and fungal classification, while the 28S rRNA gene has been found as more accurate for the classification of key groups of protozoa and helminths [43, 48–50]. While primer bias is an unavoidable limitation of amplicon studies, by sequencing two regions using different primer pairs, we identified 39 additional taxa compared to the use of the 18S rRNA V4 V5 genetic region alone. Taxa identified by both amplicons represent a high confidence set of eukaryotes and provide method validation. Low abundance organisms identified by a single amplicon may require downstream corroboration to rule out false positives. The high proportion of unclassified transITS sequences or reads with weak similarity to Amoebozoa, Parabasilids, and Platyhelminthes suggests eukaryotic diversity that remains undocumented in public databases. Application of this amplicon to further clinical and environmental samples could reveal additional diversity and provide phylogenetic resolution not possible with 18S rRNA gene studies; however, the current need for transITS fragmentation significantly curbs its potential. Recent improvements in error rates associated with long-read sequencing technologies such as those developed by Oxford Nanopore and Pacific Biosciences offer much promise in this regard. Alternative molecular tagging approaches could similarly improve the recovery of full-length amplicons and reduce spurious contig assemblies.

Severe Acute Malnutrition (SAM), characterized by wasting or nutritional edema, affects an estimated 20 million children under the age of five each year, killing up to 35% even when hospitalized [51, 52]. While previous studies have demonstrated a causal relationship between gut bacteria and SAM [53, 54], the impact of eukaryotic microbes is less well established. Given that malnutrition is a common symptom of intestinal infections by microbial eukaryotes such as *Entamoeba histolytica*, *Giardia lamblia*, and *Cryptosporidium parvum* [26, 55–57], understanding the potential contribution of eukaryotes to SAM relies on the availability of a diagnostic capable of surveying a broad diversity of eukaryotic microbes. Using a two-amplicon approach, we show a high prevalence of protozoa in a Malawian cohort of SAM patients, with two or more protozoa in 84% of samples. Our findings of colonization by multiple *Blastocystis* subtypes are in agreement with previous surveys of healthy Malawian children [25], as well as individuals from both low-income countries and Western populations [2, 3]. Trichomonads and multiple *Entamoeba* species have previously been identified in healthy Malawian children [25]. Here, we identified *Entamoeba coli* and multiple trichomonads in our malnourished cohort. The occurrence of trichomonads in the context of malnutrition warrants attention as *Tritrichomonas* spp. have been reported as rare opportunistic pathogens in humans [58–61] and shown to significantly alter the immune profile and exacerbate colitis in mice [17, 62]. Indeed, the commensal *Pentatrichomonas hominis* was previously suspected of causing gastrointestinal symptoms in two patients [63]. The prevalence of *Cryptosporidium* and *Enterocytozoon bieneusi* in the cohort is clinically relevant, as these taxa are commonly isolated from children with SAM exhibiting persistent diarrhea, and *E. bieneusi* has been associated with chronic diarrhea and intestinal malabsorption in HIV-positive adults [64–71]. Detection of nematode and cestode sequences is also pertinent as intestinal infections by a handful of species are widely implicated in malnutrition and stunting [72].

We noted a positive association between *Blastocystis* and complex carbohydrate metabolizing bacteria *Ruminococcus*, *Eubacterium*, and *Cellulosibacter*, as well as the butyrate producer, *Roseburia*—taxa typically associated with gut health [36, 54, 73–75]. Future association studies with larger cohorts may reveal whether these interactions are *Blastocystis* subtype-specific. Although *Giardia* and *Cryptosporidium*-induced bacterial dysbiosis have been reported in mice [76, 77], we found no significant associations in our cohort. We note that possible exposure of children to antibiotics prior to hospitalization may confound our analysis. As stool was collected shortly after admission, we therefore expect that effects of antibiotic treatment at the hospital had minimal impact on our findings.

Nearly half of the children in this cohort were HIV reactive. Previous studies have suggested that *Blastocystis* and *Cryptosporidium* infections are common causes of persistent diarrhea in HIV-positive children [78]. However, we found no evidence of an association between parasite carriage, HIV reactivity, and diarrhea, albeit in a relatively limited sample set, and the absence of conclusive diagnosis of infection.

While serving to illustrate the utility of our approach, the small sample size used in this study together with the complexity of the SAM phenotype and lack of samples from healthy subjects preclude us from drawing specific conclusions on the contribution of eukaryotic taxa to clinical symptoms. Applying this methodology to future cross-sectional and longitudinal case-control studies is expected to reveal clinically relevant relationships between gut eukaryotes and bacteria and help distinguish resident eukaryotes from acute infections.

During the development of our two-amplicon approach, we noted a number of limitations. First, the length of the V4 V5 genetic regions in the 18S rRNA gene exceeds current read lengths generated by Illumina sequencing platforms, resulting in incomplete sequences for many taxa, a limitation we expect to be mitigated by improvements in sequencing technology. Second, the lack of comprehensive sequence resources for rRNA genes and ITS restricts our ability to capture the full spectrum of eukaryotic diversity. Third, the recovery of human amplicons in our analyses suggests that host contamination may restrict a more comprehensive sampling of eukaryotic microbiota. Strategies such as human DNA blocking primers are needed to reduce host DNA signal. Finally, our inability to detect *Giardia*, which was detected by the Luminex pathogen panel, may be the result of primer mismatches or low abundance. Despite these limitations, we believe this two-amplicon approach provides an effective strategy for characterizing the eukaryotic microbiota of environmental samples, including those relevant to human health.

## Conclusions

To generate a diverse and robust profile of eukaryotic microbiota in the human gut, we developed a strategy based on sequencing two independent taxonomically informative genetic regions. The combined sequence information allowed us to uncover protozoa, microsporidia, helminths, and fungi, including two organisms previously associated with malnutrition, and putative relationships between the eukaryote *Blastocystis* and bacteria. We expect future applications of this method to clinical and environmental samples will begin to uncover the full diversity of eukaryotic microbes, together with their interactions with other microbiota, as well as their role in health and disease.

## Methods

### Evaluation of rRNA variable genetic regions, primer design, and validation

Sequence variability was calculated as the Shannon entropy at each position of aligned protozoan 18S (*n* = 19,427) and 28S (*n* = 1061) rRNA genes obtained from the SILVA v128 database [29]. Variable region boundaries were identified through manual inspection. To evaluate the potential for taxonomic classification, 100 copies of each variable region (≥ 30 nt) were mutated with a 1% frequency in silico and compared to reference sequences using the Burrows-Wheeler Aligner MEM (BWA-MEM) algorithm [33]. Primers were designed by manual inspection of conserved stretches in protozoan and helminth kingdoms. In silico primer binding predictions were generated using the SILVA TestProbe 3.0 tool [29], allowing 1 weighted mismatch. Experimental primer evaluation was carried out using PCR conditions described below on gDNA isolated from a panel of cultured protozoa, fungi, helminths, and a human foreskin fibroblast cell line (ATCC SCRC-1041).

### DNA extraction and sequencing

Details of clinical sample collection are provided in Additional file 2: Supplementary Methods; 100–150 mg of formed or 100 μL of loose stool was subjected to 5 min of bead beating and DNA was extracted using easyMAG (bioMerieux, Marcie-l’Etoile, France). Samples were screened for stool pathogens using the xTAG Gastrointestinal Pathogen Panel (Luminex, Austin, TX). Amplicons were generated in triplicate using the iProof polymerase (Bio-Rad Cat. #1725301, Hercules, CA) as follows: 94 °C 45 s, 56 °C 45 s, and 72 °C 1 min (18S rRNA gene V4 V5, 30 cycles, primers V4-1 forward 5′-GCGGTAATTCCAGCTC-3′ and V4-4 reverse 5′-GCCMTTCCGTCAATTCC-3′) or 3 min (transITS, 35 cycles, primers V9-9 5′-CCTGCCMTTTGTACACACC-3′ and V2-rev6 5′-GGNGCGAAAGACTMATCG-3′). Samples from 46 children produced amplicons and were used in this study. TransITS amplicons were fragmented using the Nextera kit (Illumina, San Diego, CA), and both libraries were barcoded and sequenced with MiSeq V3 (300 bp × 2) (Illumina, San Diego, CA). 16S rRNA gene V4 amplicons were generated using 515f and 806r primers, and libraries were sequenced using MiSeq V2 (150 bp × 2) (Illumina, San Diego, CA).

### Sequence processing and classification

Paired amplicon reads were quality filtered using Trimmomatic-0.36 [79], transITS and 16S rRNA gene V4 reads were merged using VSEARCH v1.11.1 [80], and all were dereplicated. In the 18S rRNA gene V4 V5 amplicon, due to low percentage of read overlap, 200 nt of high-quality forward reads were taxonomically classified using SINA v1.2.11 [81], with minimum 90% sequence identity and 1 neighbor. To confirm taxonomies, reverse reads (≥ 100 nt in length) were included in subsequent phylogenetic analyses. Paired reads from select genera were assembled into contigs with maximum 1% mismatch using the Geneious v9.02 de novo assembler [82]. Contigs composed of ≥ 100 reads (or the highest number of reads in the low abundance taxa *Gregarina*, *Tetratrichomonas*, and *Blastocystis* sp. ST6 and unknown ST) were aligned with clade representatives using MUSCLE (50 iterations), and optimal gap trimming was performed using trimAl v1.2 [83, 84]. Bayesian phylogenies were constructed using MrBayes [85], with the HKY85 substitution model, 1 million chain length, four heated chains, a burn-in of 250,000, and four gamma categories. Trees were visualized using FigTree v1.4.3 [86], and branches with posterior probability values ≤ 0.7 were collapsed.

TransITS reads were classified using BWA-MEM [33] based on the closest sequence match (≥ 97% sequence identity, ≥ 70% coverage) in a custom database. The database was built using 18S and 28S rRNA genes obtained from SILVA, UNITE, and RFAM databases [29, 33, 87, 88], as well as protozoan and helminth rRNA genes captured from the NCBI nucleotide database using the search terms “*ITS1*,” “*ITS2*,*”* and “*28S*.” Reference sequences were trimmed with cutadapt v1.12 [89], using amplicon primer sequences. Positional analysis of reads was performed by mapping merged reads to known reference sequences, or where no references were available, to contig consensus sequences generated through de novo sequence assembly, using Geneious v.9.02.

Unclassified amplicon sequences represented by ≥ 500 reads were submitted for a BLAST [90] search of the NCBI nucleotide database (downloaded Dec 1, 2017) and assigned the taxonomy of the top hit (≥ 98% sequence identity and coverage). *Blastocystis* subtypes were identified by ≥ 98% sequence identity to their respective genomes; equivalent scores to more than one subtype were assigned “*Blastocystis* unknown ST.” All reference gene and genome accessions are provided in Additional file 1: Table S5.

16S rRNA gene amplicons were clustered de novo to 97% using the VSEARCH cluster_fast algorithm, and taxonomies were assigned using the RDP classifier (release 11, training set 16) [91].

### Analysis of microbiota composition

Microbiota composition and alpha diversity analyses were carried out in R 3.4 using Phyloseq 1.20.0 [92] and ggplot2 2.2.1 [93] packages. Similarity of eukaryotic 18S rRNA gene V4 V5 and transITS profiles were evaluated using taxon co-occurrence and Bray-Curtis compositional dissimilarity. Distances between samples, based on genus co-occurrence, were calculated using Hamming distances in the e1071 1.6-8 package [94] as follows. OTU tables were transformed to binary matrices, using a range of 5–100 minimum read thresholds to indicate the presence of taxa, and numbers of co-occurring taxa were scored. Significance was tested between the distribution of Hamming distances scores of profiles of the same patients compared to different patients using the two-sample Kolmogorov-Smirnov test. Bray-Curtis distances were calculated using the composition of genera identified in at least 20% of 18S rRNA gene V4 V5 and transITS datasets. Significance was tested for within-patient and between-patient comparisons using the Wilcoxon rank sum test.

Putative associations between eukaryotic and bacterial taxa were identified using taxon co-occurrence, differential abundance analysis, and sparse partial least squares discriminant analysis. Co-occurrence of bacterial and eukaryotic taxa in patient samples was calculated using Hamming distances as above, using a minimum of five reads to designate occurrence. Taxa occurring in < 20% or > 80% of samples were removed to reduce spurious associations. Distances were clustered and plotted using the hclust and heatmap.2 functions in the gplots 3.0.1 R package [95]. Bacterial differential abundance analysis was carried out with ALDEx2 v1.10.0 [96], using centre log-transformed data, prefiltered for bacterial taxa present at ≥ 2 reads in ≥ 2 patients. Abundance was compared in samples positive and negative for eukaryotes with enough variance for analysis (*Blastocystis*, *Cryptosporidium*, *Giardia*, and *Enterocytozoon*) or HIV reactivity, and *p* values were calculated using the Wilcoxon rank sum test with Benjamini-Hochberg correction. Tests were carried out using minimums of 2, 5, 50, and 100 reads in patient samples to designate the presence of eukaryote, and *p* values < 0.05 in at least two tested conditions were deemed significant. Sparse partial least squares discriminant analysis (sPLS-DA) was used to evaluate relationships between patient samples positive and negative for *Blastocystis*, based on bacterial composition. Data were prefiltered for bacterial genera present at ≥ 5 reads in ≥ 10% of patients, and abundance was normalized using cumulative sum scaling in the MixOmics 6.2.0 [97] and MetagenomeSeq [98] packages. The model was evaluated through tenfold cross-validation, and classification error rates averaged over 50 repetitions. Ordered multinomial logistic regression was used to explore relationships between clinical features and the four eukaryotic organisms, binned into undetected, low, medium, and high detection.

## Additional files

Additional file 1: **Table S1.** Accuracy of taxonomic assignments of 18S and 28S rRNA variable genetic regions. Related to Fig. 1. **Table S2.** Primer characteristics and TestProbe3.0 results. Related to Fig. 2. **Table S3.** Amplicon sequencing statistics. **Table S4.** Read counts for eukaryotic microbes detected by two amplicon methods in patients suffering from SAM. Related to Fig. 3. **Table S5.** Genome and gene accessions used for comparisons with stool sample DNA and phylogenetic trees. **Table S6.** Differential abundance analysis of bacteria based on of *Blastocystis* carriage, using ALDEx2. Related to Fig. 5. **Table S7.** Association between eukaryote carriage or HIV reactivity and bacterial diversity. Related to Fig. 5. **Table S8.** Association between eukaryote abundance and clinical characteristics of patients hospitalized for SAM. (XLS 162 kb)

Additional file 2: **Figure S1.** Eukaryotic microbiota of Malawian cohort suffering from SAM. a Relative sequence abundances of all eukaryotic phyla identified in the V4 V5 amplicon of the 18S rRNA gene (*n* = 44). Columns represent individual patients, and samples are numbered for comparison with DNA gels. b PCR validation of presence of *Pentatrichomonas* and *Cryptosporidium* using published primers Th4, Th5, Cr254F and CR323R (Crucitti et al. 2004; Bruijnesteijn et al. 2009), and an *Entamoeba coli* specific forward primer 5′-GGTTTCTAATATCAACAGGCTAC-3′ to 18S rRNA gene FR686446 with 18S-V9-15_R. NT, no template control; samples marked with an asterisk are not expected to be positive. Related to Fig. 3 a. **Figure S2.** Differential composition analysis of bacterial OTUs in Malawian samples. Plots were generated with the ALDEx2 R-package, and show OTU fold-change versus median abundance *(left)* and OTU fold-change between versus within conditions *(right)*. No significant compositional changes in OTUs were found by Wilcoxon rank test. Tests compare subjects positive and negative for a, *Cryptosporidium* (18S rRNA V4 V5 amplicon), b, *Enterocytozoon* (transITS amplicon), c, *Giardia* or d, HIV. *Cryptosporidium* and *Enterocytozoon* presence are defined as a minimum of 5 amplicon reads, and *Giardia* and HIV infection status were determined by clinical diagnostic tests. Related to Fig. 5b. Supplementary Methods. Supplementary information is included on clinical sample collection. (PDF 1241 kb)

## Abbreviations

SAM: Severe Acute Malnutrition
V: Variable region
