# Supplementary material for: Design and application of a novel two-amplicon approach for defining eukaryotic microbiota
Source: Microbiome. 2018 Dec 20;6:228. doi: 10.1186/s40168-018-0612-3 (PMC6302507; doi:10.1186/s40168-018-0612-3)
Supplement: Supplementary file 2 — Figure S1. Eukaryotic microbiota of Malawian cohort suffering from SAM. a Relative sequence abundances of all eukaryotic phyla identified in the V4 V5 amplicon of the 18S rRNA gene (n = 44). Columns represent individual patients, and samples are numbered for comparison with DNA gels. b PCR validation of presence of Pentatrichomonas and Cryptosporidium using published primers Th4, Th5, Cr254F and CR323R (Crucitti et al. 2004; Bruijnesteijn et al. 2009), and an Entamoeba coli specific forward primer 5′-GGTTTCTAATATCAACAGGCTAC-3′ to 18S rRNA gene FR686446 with 18S-V9-15_R. NT, no template control; samples marked with an asterisk are not expected to be positive. Related to Fig. 3 a. Figure S2. Differential composition analysis of bacterial OTUs in Malawian samples. Plots were generated with the ALDEx2 R-package, and show OTU fold-change versus median abundance (left) and OTU fold-change between versus within conditions (right). No significant compositional changes in OTUs were found by Wilcoxon rank test. Tests compare subjects positive and negative for a, Cryptosporidium (18S rRNA V4 V5 amplicon), b, Enterocytozoon (transITS amplicon), c, Giardia or d, HIV. Cryptosporidium and Enterocytozoon presence are defined as a minimum of 5 amplicon reads, and Giardia and HIV infection status were determined by clinical diagnostic tests. Related to Fig. 5b. Supplementary Methods. Supplementary information is included on clinical sample collection. (PDF 1241 kb) [file 40168_2018_612_MOESM2_ESM.pdf]

Additional file 2

Supplementary figures

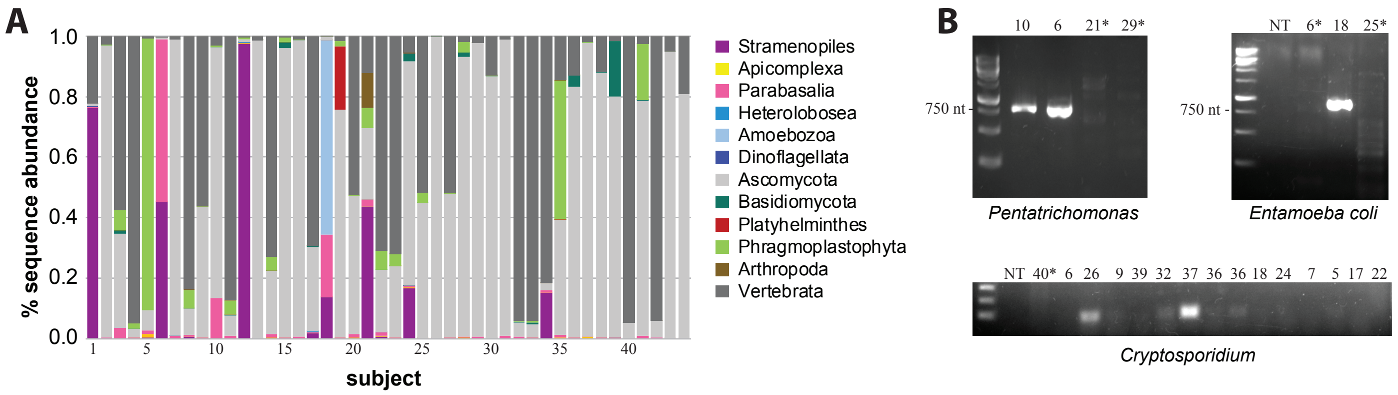

Figure S1. Eukaryotic microbiota of Malawian cohort suffering from SAM. a Relative sequence abundances of all eukaryotic phyla identified in the V4V5 amplicon of the 18S rRNA gene (n=44). Columns represent individual patients. Samples are numbered for comparison with DNA gels. **b** PCR validation of presence of *Pentatrichomonas* and *Cryptosporidium* using published primers Th4, Th5, Cr254F and CR323R (Crucitti *et al*. 2004; Bruijnesteijn *et al*. 2009), and an *Entamoeba coli* specific forward primer 5′-GGTTTCTAATATCAACAGGCTAC-3′ to 18S rRNA gene FR686446 with 18S-V9-15_R. NT, no template control; samples marked with an asterisk are not expected to be positive.

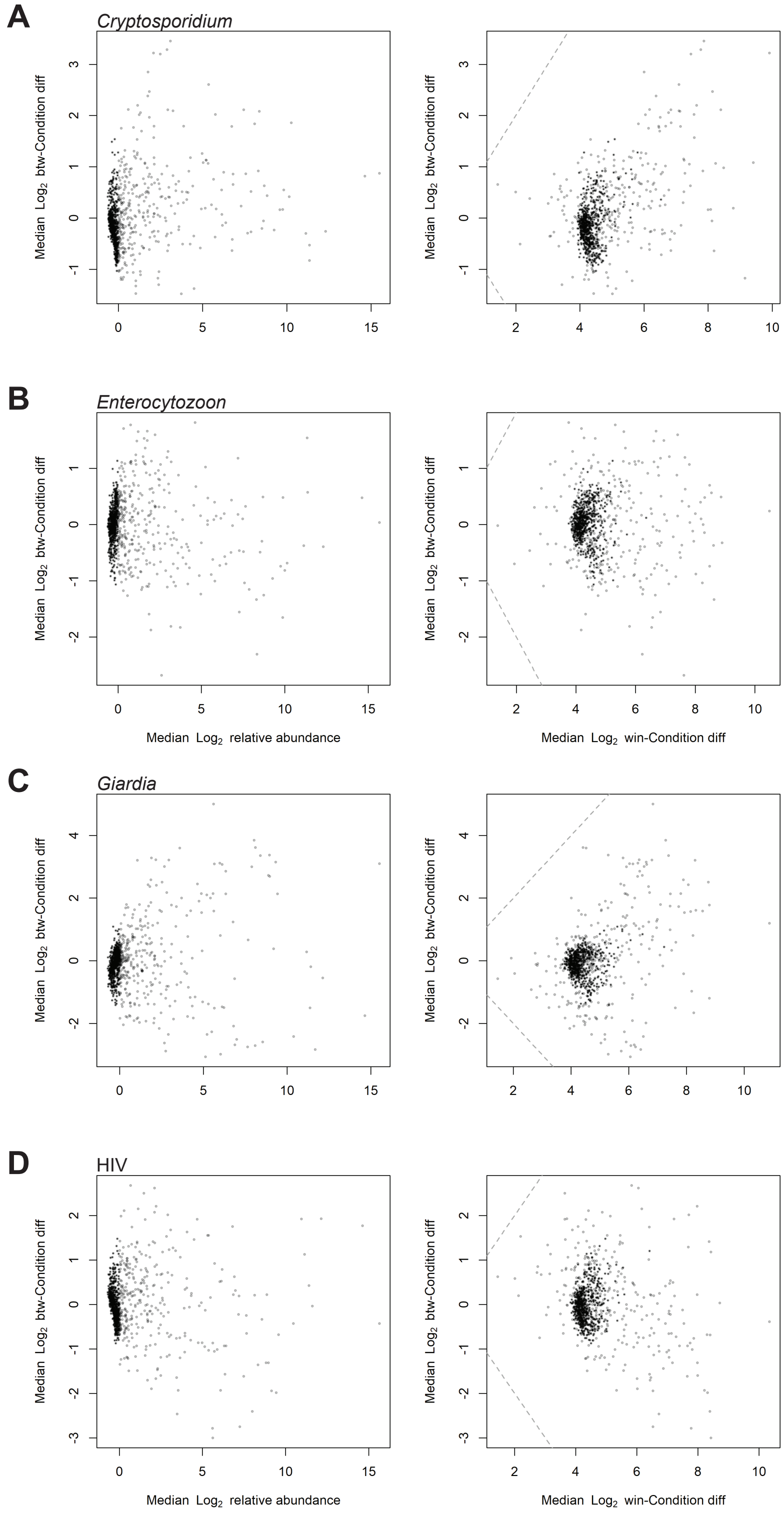

Figure S2. Differential composition analysis of bacterial OTUs in Malawian samples. Plots were generated with the ALDEx2 R-package, and show OTU fold-change versus median abundance *(left)* and OTU fold-change between versus within conditions *(right)*. No significant compositional changes in OTUs were found by Wilcoxon rank test. Tests compare subjects positive and negative for **a,** *Cryptosporidium* (18S rRNA gene V4V5 amplicon), **b,** *Enterocytozoon* (transITS amplicon), **c,** *Giardia* or **d,** HIV. *Cryptosporidium* and *Enterocytozoon* presence are defined as a minimum of 5 amplicon reads, and *Giardia* and HIV infection status were determined by clinical diagnostic tests.

Supplementary Methods

Clinical data and stool collection

Patient samples, with informed consent, were drawn from a previous randomized clinical trial (www.isrctn.com/ISRCTN13916953) [3, 4] following 90 children with complicated SAM, 6-60 months of age, admitted to the Queen Elizabeth Central Hospital in Blantyre, Malawi. Complicated SAM was defined as <-3 weight-for-height z-scores (WHZ) and/or mid-upper arm circumference <115mm or nutritional bilateral pitting edema with medical complications as defined by WHO guidelines [5]. Children with severe hemodynamic instability, haematocrit levels ≤ 15%, severe neurological symptoms or those admitted for malnutrition within the previous year were excluded. Anthropometric, demographic and clinical data were collected at hospital admission. HIV reactivity was determined by rapid antibody testing. Stool samples were collected within 24 h of admission, immediately cooled and frozen at -80°C. Study approval was obtained from the Research Ethics Board at The Hospital for Sick Children (Toronto, Canada) and the College of Medicine Research Ethics Committee at the University of Malawi (Blantyre, Malawi).

Supplementary Material References

1. Crucitti T, Abdellati S, Ross DA, Changalucha J, Dyck E, Buve A: **Detection of Pentatrichomonas hominis DNA in biological specimens by PCR**. *Lett Appl Microbiol* 2004, **38**(6):510-516.
2. Bruijnesteijn van Coppenraet LE, Wallinga JA, Ruijs GJ, Bruins MJ, Verweij JJ: **Parasitological diagnosis combining an internally controlled real-time PCR assay for the detection of four protozoa in stool samples with a testing algorithm for microscopy**. *Clin Microbiol Infect* 2009, **15**(9):869-874.
3. Versloot CJ, Voskuijl W, van Vliet SJ, van den Heuvel M, Carter JC, Phiri A, Kerac M, Heikens GT, van Rheenen PF, Bandsma RHJ: **Effectiveness of three commonly used transition phase diets in the inpatient management of children with severe acute malnutrition: a pilot randomized controlled trial in Malawi.** *BMC pediatrics* 2017, **17**(1):112.
4. Attia S, Versloot CJ, Voskuijl W, van Vliet SJ, Di Giovanni V, Zhang L, Richardson S, Bourdon C, Netea MG, Berkley JA *et al*: **Mortality in children with complicated severe acute malnutrition is related to intestinal and systemic inflammation: an observational cohort study.** *American Journal of Clinical Nutrition* 2016, **104**(5):1441-1449.
5. WHO. In: *Guideline: Updates on the Management of Severe Acute Malnutrition in Infants and Children.* Geneva; 2013.
